# The Structure of *Helicobacter pylori* HP0310 Reveals an Atypical Peptidoglycan Deacetylase

**DOI:** 10.1371/journal.pone.0019207

**Published:** 2011-04-29

**Authors:** Md Munan Shaik, Laura Cendron, Riccardo Percudani, Giuseppe Zanotti

**Affiliations:** 1 Department of Biological Chemistry, University of Padua, Padua, Italy; 2 Venetian Institute of Molecular Medicine (VIMM), Padua, Italy; 3 Department of Biochemistry and Molecular Biology, University of Parma, Parma, Italy; National Institute for Medical Research, Medical Research Council, London, United Kingdom

## Abstract

Peptidoglycan deacetlyase (HP0310, HpPgdA) from the gram-negative pathogen *Helicobacter pylori*, has been indicated as the enzyme responsible for a peptidoglycan modification that counteracts the host immune response. HpPgdA has been cloned, purified and expressed in good yield in *E. coli*. It has been crystallized, its structure determined and activity tests *in vitro* performed. The enzyme, which belongs to the polysaccharide deacetylases protein family, is a homo-tetramer. The four polypeptide chains, each folded into a single domain characterized by a non-canonical TIM-barrel fold, are arranged around a four-fold symmetry axis. The active site, one per monomer, contains a heavy ion coordinated in a way similar to other deacetylases. However, the enzyme showed no *in vitro* activity on the typical polysaccharide substrates of peptidoglycan deacetylases. In striking contrast with the known peptidoglycan deacetylases, HpPgdA does not exhibit a solvent-accessible polysaccharide binding groove, suggesting that the enzyme binds a small molecule at the active site.

## Introduction

Peptidoglycan, which is one of the constituents of the protective barrier of gram-negative bacteria, at the same time represents one of the main targets of the innate immune system of the host [1]. Some peptidoglycan components are recognized by specific receptors of human cells, Nod1 and Nod2, which initiate the immune response by activating the NF-kB pathway [2], [3]. In order to escape the recognition by the host receptors, some bacteria have developed a mechanism of chemical modification of peptidoglycan, in particular through N-deacetylation [1]. In *Helicobacter pylori*, the gram-negative pathogen that affects about half of the human population, this task is accomplished by a peptidoglycan deactelyase coded by gene *hp0310* [4], [5]. It was in fact demonstrated that the HP0310 protein was over-expressed under strong oxidative stress conditions and that peptidoglycan of a HP0310 knock-out mutant presented larger acetylation compared to wild-type, along with increased susceptibility to lysozyme degradation. Moreover, expression of HP0310 was induced when *H. pylori* was held in contact with macrophages and a significant increase of IL-10 and TNF-α was observed in mutant-infected mice compared to wild-type.

According to all existing evidence, HP0310 was recognized as a *H. pylori* peptidoglycan deacetylase (HpPgdA). HpPgdA is a soluble protein of 293 amino acids, which has limited sequence similarity with enzymes of the polysaccharide deacetylase family (pfam01522). Among the proteins of the family that have been well characterized is the peptidoglycan deacetylase from *Streptococcus pneumoniae* (SpPgdA), a Zn-dependent enzyme [6]. It is significantly longer than HpPgdA (463 amino acids) and it shares less than 19% identity with our protein. Moreover, the *H. pylori* enzyme apparently lacks the conserved metal-binding motif [5]. Nevertheless, reliable HpPgdA homologues can be found in several pathogenic bacteria. For the above reasons, HpPgdA was proposed to be the prototype of a new family of polysaccharide deacetylases [5]. In addition, in a proteomic analysis of a *H. pylori* strain adapted *in vivo* to induce gastric adenocarcinoma in rodent models (7.13 strain), the loss of a functional HpPgdA resulted in higher levels of CagA translocation compared to the non-carcinogenic strain. This observation suggests that peptidoglycan alteration by HpPdgA could affect the composition of substrates translocated by the *cag* secretion system [7].

If a deacetylation activity has been already established for the protein, the specific substrate of HpPgdA has yet to be identified. It is also possible that HpPgdA is active on substrates other than peptidoglycan, for example lipopolysaccharides or membrane anchored proteins. In order to elucidate the structural features of the prototype of a new family of deacetylase and to establish a possible enzymatic mechanism, peptidoglycan deacetlyase (HP0310) from *H. pylori* was cloned, expressed and purified in good yield in *E. coli*. The enzyme was crystallized, its structure determined and *in vitro* activity tests were performed.

## Results

### The overall model

The crystal structure, despite the relatively limited resolution, 2.57 Å, appears quite reliable: the electron density for the protein main chain atoms is clearly visible from residue 2 to 291, without gaps and with a good stereochemistry. Only the N-terminal methionine and two residues at the C-terminus are missing in the crystallographic model.

**Figure 1:**
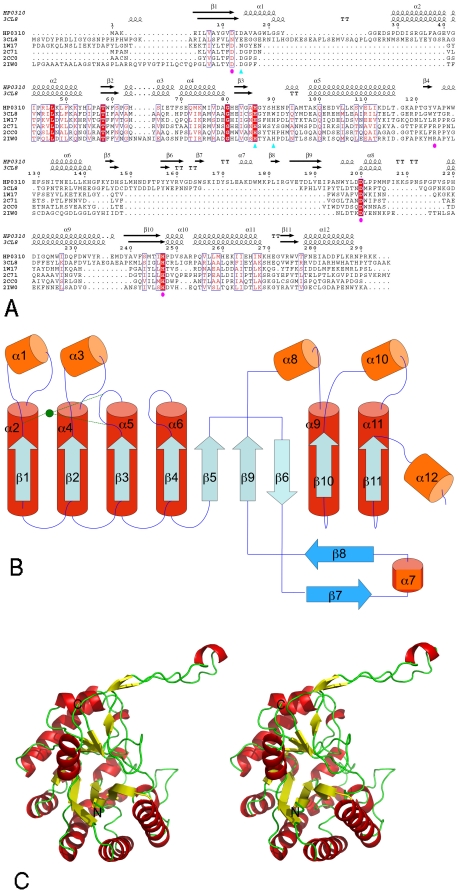
Topology and structure of HpPgdA monomer. A) Amino acid sequence of HP3010 protein aligned with PuuE allantoinase from *P. fluorescens* (Protein Data Bank code 3CL8), peptidoglycan deacetylase from *B. subtilis* (Protein Data Bank code 1W17), acetyl xylan esterases from *C. thermocellum* (Portein Data Bank code 2C71) and from *S. lividans* (Protein Data Bank code 2CC0), and chitin deacetylase from *C. lindemuthianum* (Protein Data Bank code 2IW0). Secondary structure elements deriving from Protein Data Bank coordinates are drawn over the alignment. Residues previously reported to be involved in metal-binding or in catalysis are denoted by cyan arrowheads (metal-binding) and magenta circles (catalysis). B) Topology diagram of HpPgdA. Helices and strands of the modified TIM barrel are in red and cyan, respectively; helices and strands surrounding the barrel in orange and light blue, respectively. The location of heavy ion is indicated by a green filled circle. C) Stereo view of a Cartoon of HpPgdA monomer. α-helices are in red, β-strands in yellow, others in green. N and C-terminus are labeled. (This and the following drawing were done using the Pymol program [31].

**Figure 2:**
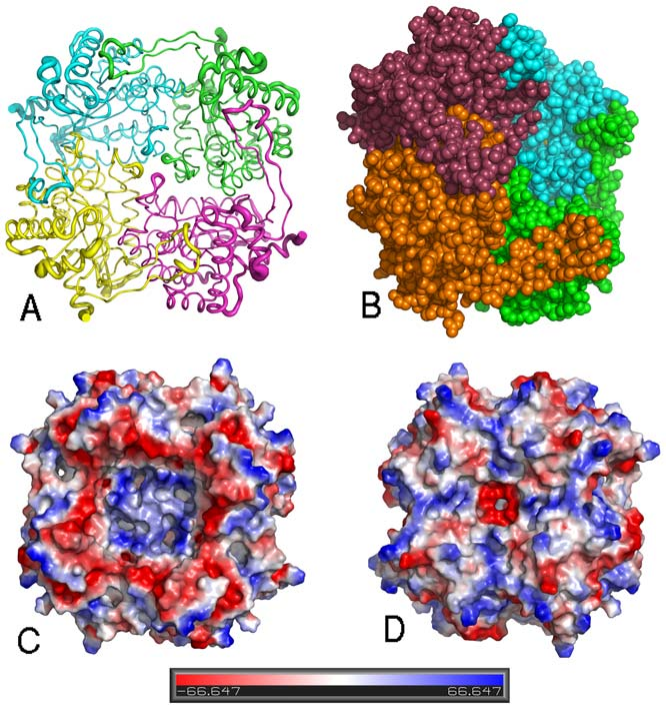
The HpPgdA tetramer. A) Ribbon drawing of the tetrameric assembly of HpPgdA. The view is approximately perpendicular to the molecular four-fold axis. The diameter of the ribbon tube is proportional to the thermal parameters of the atoms. B) van der Waals model of HpPgdA tetramer. Each monomer is colored differently, so that it is possible to se the long arm protruding from the core of one monomer to embrace the nearby one in the tetramer. The orientation is slightly rotated with respect to Fig. 2A, so that one heavy ion (yellow) is partially visible in one subunit. C) And D) Qualitative electrostatic potential surface of HpPgdA tetramer. In the left picture the orientation is similar to that of Fig. 2A and represents the side from which the active site is partially accessible. The ion is totally concealed under the surface. The picture on the right shows the opposite face of the tetramer.

The secondary structure elements of the monomer are illustrated in Fig. 1A, and their topology and tertiary structure organization in Fig. 1B and 1C. The polypeptide folds into a single domain, characterized by a non-canonical TIM-barrel fold [8]: nine ß-strands, labeled from 1 to 6 and from 9 to 11, are arranged in a central barrel surrounded by six α-helices (labeled α-2, 4, 5, 6, 9, 11). Helices α -1, α-3, α-7, α-8, α-10 α-12 surround the barrel on the two sides. The topology (Fig. 1B) can be compared with those of peptidoglycan deacetylase from *S. pneumoniae* (SpPgdA, see Fig. 2A of paper [6]) or from *B. subtilis* (PdaA [9]). The more significant difference from the other members of the family is represented by a long arm of about 25 residues, from 161 to 185, which extends away from the molecular core and embraces a nearby enzyme monomer, strengthening the quaternary organization of the protein.

### Quaternary structure

Four monomers are present in the asymmetric unit, arranged around a four-fold rotation axis (Fig. 2). The enzyme in solution is also a tetramer, as confirmed by gel-filtration experiments (Figure S2). The four monomers do not present significant conformational differences, and in fact non-crystallographic restraints were applied through all the refinement cycles. The root mean square deviation (r.m.s.d.) between equivalent main chain atoms of different monomers is in the range 0.27–0.28 Å. Interactions among monomers are both hydrophobic and hydrophilic: there are 29 hydrogen-bond interactions between two adjacent monomers, along with several hydrophobic contacts. The surface buried after tetramer formation is 11,420 Å^2^, which represents nearly one third of the sum of the area of the four isolated monomers, 36,858 Å^2^. The most relevant stabilization of the quaternary organization of the protein is the long arm that protrudes from one monomer to embrace the other.

### Structure comparison

An exhaustive search with DALI server [10] shows that the two three-dimensional structures most similar to HpPgdA are a *bona fide* allantoinase [11] from *Pseudomonas aeruginosa* (PDB ID 1z7a, Chang, Skarina, Savchenko, Edwards, Joachimiak, Z-score 30.5, r.m.s.d. from our structure for 300 amino acids 1.9 Å) and PuuE allantoinase from *Pseudomonas fluorescens* (PDB ID 3cl8 [11], Z-score 30.3, r.m.s.d. for 300 superimposed amino acids 2.0 Å). The latter was in fact used as a template to build the model for the molecular replacement. The most relevant differences from our structure are represented by the long arm that in HpPgdA protrudes from the monomer core, by a loop region (205–217) connecting helices α-7 to α-8, which is definitely longer in HpPgdA, and by the loop connecting helices α-1 to α-2, which in HpPgdA includes only five residues, whilst in PuuE is about 27 residues long. It must be considered that the latter area in PuuE is close to the active site and some of the residues may be involved in substrate recognition.

Some structural similarities are also observed with the catalytic domain of 4- α-glucanotransferase (PDB ID 1k1x [12]) and α-amilase (PDB ID 2b5d [13]). Members of the same enzymatic family, such as SpPgdA from S. *pneumoniae* (PDB ID 2c1g [6]) and PdaA from *B. subtilis* (PDB ID 1w17 [9]) are structurally less similar, consistent with a lower sequence identity.

### The active site

**Figure 3:**
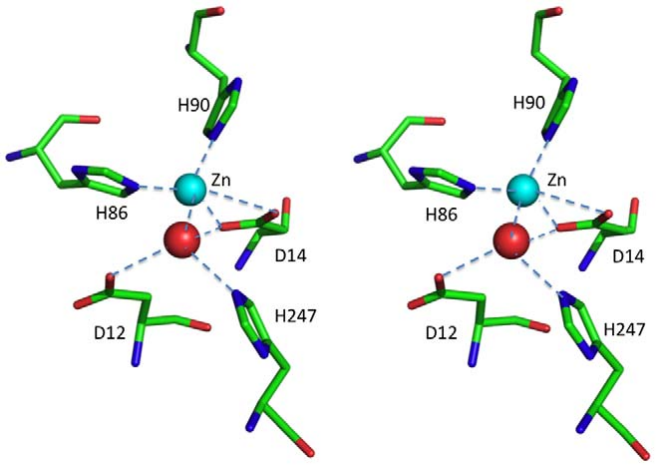
HpPgdA active site. The Zn ion (cyan) is coordinated by Nε of His 90 (distance 2.06 Å), Nε of His 86 (2.12 Å), Oδ1 and Oδ2 of Asp 14 (2.21 Å and 2.98 Å), a water molecule (red sphere, 2.08 Å). Distances between the latter and Oδ2 of Asp 12, Nε of His 247 and Oδ1 of Asp 14 are 2.68 Å, 2.60 Å and 2.99 Å, respectively.

The putative active site of *H. pylori* peptidoglycan deacetylase is represented by a heavy ion coordinated to the Nε nitrogen atoms of His 86 and 90 and to the two oxygen atoms of Asp 14. A fourth coordination site is occupied by a water molecule. The latter is held in place by H-bonds with His 247, Asp 12 and 14 (Fig. 3). Since a similar situation has been observed in two other mono-nuclear Zn-dependent deacetylases, polysaccharide deacetylase from *B. subtilis* (PdaA) [9] and peptidoglycan GlcNac deacetylase from *S. pneumoniae* (SpPgdA) [6], the ion was assumed to be a Zn. In both cases, the main difference from our structure is represented by the accessibility to the active site: in HpPgdA the presence of helix α1, the longest helix α2 and the fact that the loop connecting helices α7 to α8 protrudes towards the active site opening, makes it poorly accessible. Moreover, some bulky side chains, in particular Trp 127 and 196, Tyr 23 and Leu 201 obstruct the access to the ion (Fig. 2B).

### Activity tests

**Figure 4:**
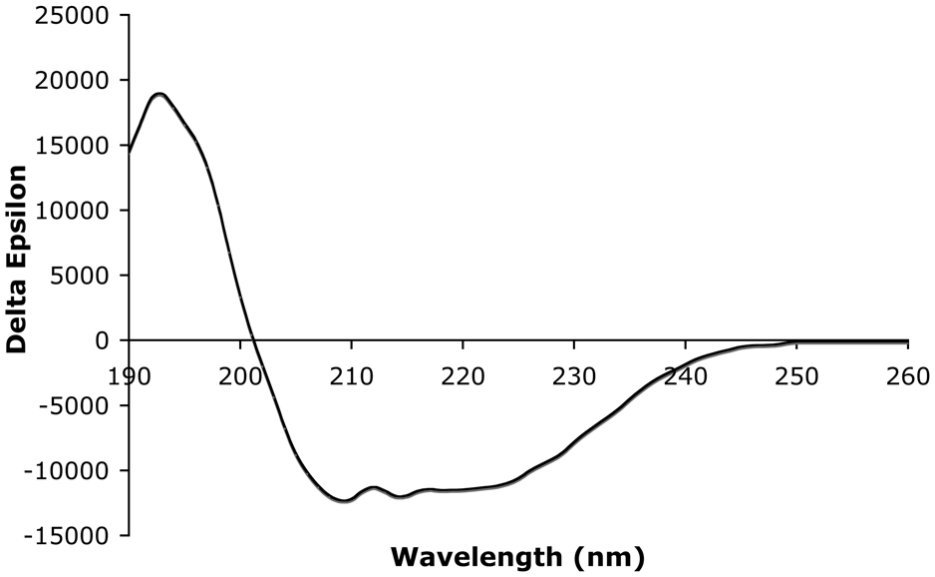
Circular dichroism spectrum of HP0310. Spectrum was measured with J-715 spectropolarimeter (JASCO, Corporation) at 298 K. 10 runs were accumulated with 1 mg/ml protein (Tris 3 mM, NaCl 10 mM, pH 8.0) in a 0.2 mm path cuvette in the wavelength interval 190–260 nm. The CD spectrum was rescaled with respect to a standard solution containing the buffer and the molar ellipticity calculated. The CD spectrum was deconvoluted with software “CD Spectra Deconvolution” (CDNN 2.1) and the secondary structure predicted (36.6% α-helix, 15.6% β-sheet and 47.6% other).

In order to address the substrate specificity of HpPgdA, single components of peptidoglycan were tested in solution, in particular N-acetyl glucosamine (GlcNAc), and chitotriose GlcNAc3. All the activity tests were performed using the standard conditions used for other polysaccharide deacetylases. In order to check that the protein we have purified is properly folded in solution, a circular dichroism spectrum, showing the expected content of α-helix and β-strands, was performed (Fig. 4). The gel-filtration experiment shows that the protein elutes as a single tetrameric species (see Figure S2). Since no activity was detected, other acetylated amines possibly present in *H. pylori* were tested: N-acetyl Putrescine, N-acetyl Spermidine, N-acetyl cadaverine, and some N-acetyl dipeptides (Ac-D-Ala-D-Ala-OH, Ac-D-Ala-D-Ala-OCH3, Ac-D-Ala-L-Ala-OH, Ac-D-Ala-L-Ala-OCH3). These substrates were suggested by comparative gene cluster analyses showing that genes homologous to HpPgdA are physically associated with genes encoding polyamine importers and genes encoding D-Ala-D-Ala peptidases (Figure S1). Peptide chains containing D-amino acids are ubiquitous peptidoglycan components, while polyamines have been reported to be present in the peptidoglycan of some bacteria [14]. Finally, allantoin was also tested, given the close structural similarity of HpPgdA to PuuE allantoinase. The enzyme, however, was found inactive for all of these compounds at all pH tested (6, 7, and 7.5).

### Phylogenetic relationship with other polysaccharide deacetylases

**Figure 5:**
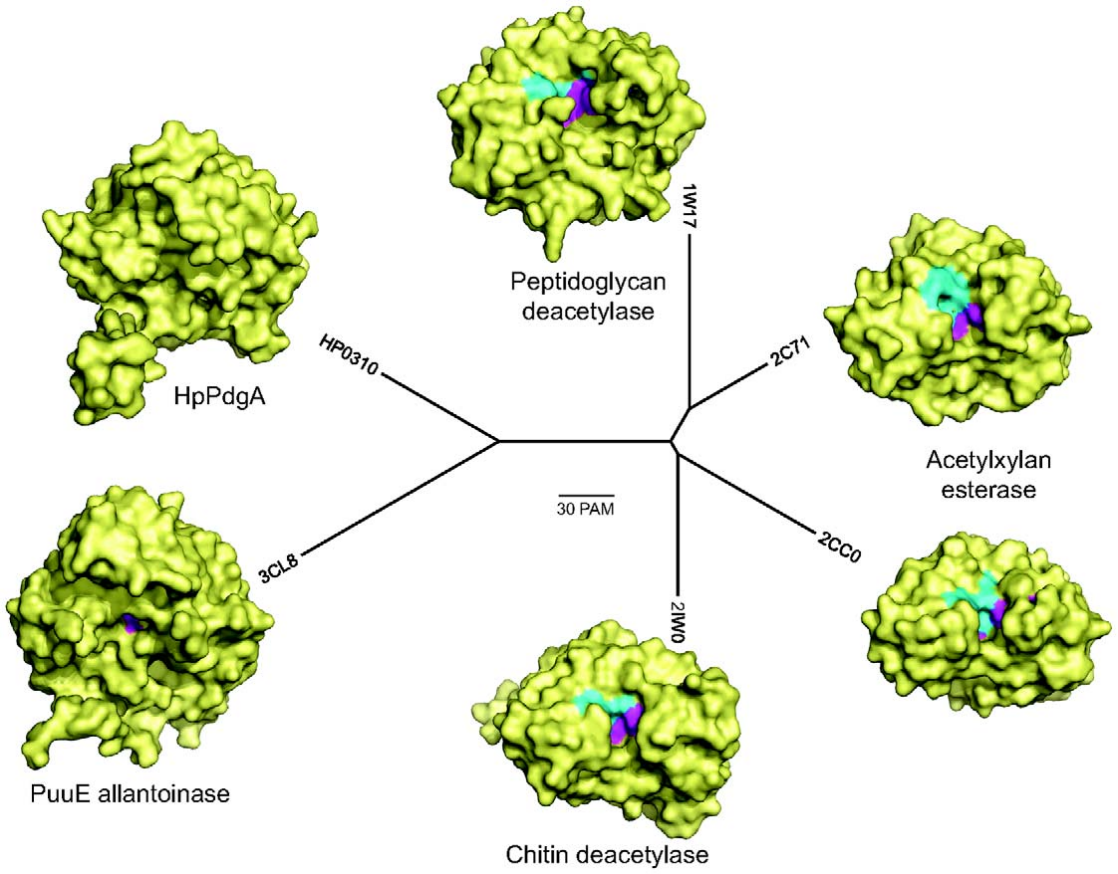
Phylogenetic and structural comparison of the polysaccharide deacetylase family. Unrooted tree showing the phylogenetic relationship of HpPgdA with other polysaccharide deacetylase proteins. Branches are proportional to genetic distance as indicated by the scale bar representing point-accepted mutations (PAM). Proteins are indicated by PDB codes. Monomeric structures are shown in the same orientation (from the top of the barrel) as surfaces, with solvent-accessible residues involved in metal-binding or catalysis (see Fig. 1A) highlighted in cyan and magenta, respectively.

Polysaccharide deacetylase is a very large and functionally variegated protein family to which HpPgdA belongs. The structure of several members of the family is known from structural genomics and dedicated studies. We selected representative members of the polysaccharide deacetylase family with known structure (see Fig. 1A) for a phylogenetic comparison with HpPgdA (Fig. 4). Most members of the family act on polysaccharide substrates and are involved in cell wall modifications such as peptidoglycan deacetylases, chitin deacetylases, xylan esterases. A common feature of polysaccharide deacetylases that recognize oligosaccharide substrates is the presence of a large groove at the top of the barrel, with the residues involved in metal binding or catalysis typically exposed at the surface of the active site (Fig. 5). Consistent with the previously reported structural similarities, the phylogenetic analysis shows that HpPgdA has a distant relationship with proteins that bind polysaccharides and a closer relationship with PuuE allantoinase. This member of the polysaccharide deacetylase family has a considerably reduced binding cavity and recognizes a small substrate molecule [11]. Similarly to PuuE, additional structural elements located at the top of the barrel in HpPgdA obstruct the access to the active site (Fig. 5). At variance with PuuE, however, HpPgdA has the ability to bind a metal ion at the active site, as observed in most members of the family (see Fig. 1A).

## Discussion

HP0310 has been demonstrated to be a peptidoglycan deacetylase in a test performed on a crude extract of peptidoglycan from *H. pylori* [5], despite the activity on a specific peptidoglycan component was not identified. Subsequent experiments on a PgdA mutant [4], [5] have supported this finding, though indirectly. The three-dimensional structure of the enzyme is compatible with this function: the putative active site of HpPgdA contains an ion, possibly Zn^2+^, coordinated in the same way as chitin deacetylase from *C. lindemuthianum* and similar to that of acetyl xylan esterases from *C. thermocellum* and *S. lividans*. Residues involved in catalysis are also conserved (Fig. 1A). The main difference of our enzyme from other members of the family is the accessibility to the active site, that fully justifies the absence of enzymatic activity on large substrates. The presence in HP0310 of two additional helices, α-1 and α-2, and its tetrameric assembly, in particular the long arm protruding from a nearby subunit, obstruct the access to the active site. Other enzymes of the family are monomeric or even dimeric, but in the latter case the active site is far from the dimerization side, as in the *S. lividans* xylan esterase. In addition, some bulky residues, in particular Trp 127, interpose between the ion bound and the solvent. This suggests that the access of the substrate to the active site must be accompanied by at least a movement of the side chain of Trp127 or possibly by a conformational rearrangement of the loop containing residues Trp 127 and 128. It is unlikely, however, that these structural rearrangements can generate an active site cavity large enough to accommodate a large polysaccharide. On the other hand, typical polysaccharide deacetylases do not show activity with monomeric substrates. For example, Chitin deacetylases need at least a chito-oligosaccharide trimer for activity but prefer longer multimers [15], [16].

The lack of enzymatic activity on isolated N-acetyl polysaccharides we observed for HpPgdA and the reported deacetylase activity on crude peptidoglycan extracts from *H. pylori* [5] could be reconciled if HpPgdA recognizes a peptidoglycan component that is different from the polysaccharide moiety. From the structure analysis, it appears more likely that HpPgdA binds a small molecule at the active site, though we cannot exclude that a significant conformational modification in the ternary and quaternary structure of the enzyme can take place in order to accommodate a large substrate. An enzyme with a similar tetrameric arrangement and a close phylogenetic relation with HpPgdA is PuuE allantoinase, whose natural substrate is in fact a small cyclic imide.

Another feature that distinguishes HpPgdA and its close homologues from classical peptidoglycan deacetylases is the lack of a signal peptide for secretion in the periplasmic space, a characteristic that is expected for an enzyme exclusively acting on cell-wall peptidoglycan. In proteomic analysis, HpPgdA has been found moderately enriched (1.8 ratio) in the extracellular fraction compared to the soluble cell-associated samples [17] suggesting an additional cytoplasmic role for the enzyme.

### Conclusions

Bacterial peptidoglycan is a complex molecule that undergoes significant processing [18]. Many fine details of peptidoglycan modifications and of the enzymes active in editing the cell-wall structure are still poorly understood. We have presented evidence that the HpPgdA substrates are not the N-acetylated polysaccharides recognized by the typical peptidoglycan deacetylases. The determination of the HpPgdA structure provides valuable information for the precise identification of its role in peptidoglycan metabolism and for a deeper understanding of those cell-wall modifications that affect the ability of bacteria to evade the host-immune system response.

## Materials and Methods

### Cloning, expression, purification

The HP0310gene was amplified by PCR from genomic *H. pylori* G27, using the primers 5′-CACCATGGCAAAAGAAATTTTAGTGGC-3′ (forward, Topoisomerase recognition site underlined) and 5′-TTACTATTTTTTTCTAGGGTTTCGTTTTAAG-3′ (reverse). It was cloned into the pET151 vector (pET151; Invitrogen) in frame with an N-terminal His-tag flanked by a TEV proteolysis site, using a TOPO® Cloning kit by Invitrogen. *E. coli* BL21(DE3) cells, harboring the pET151-HP0310 plasmid, were grown in LB medium supplemented with 100 µg/mL ampicillin and the protein expression induced by 1 mM isopropyl-β-D-thiogalactopyranoside (IPTG). The bacterial pellet was resuspended in 30 mM Tris pH 8.0, 150 mM NaCl; cells lysis was performed by a two-step method, via incubation with lysozyme (1 mg/ml, 1 h, 4°C) and sonication. The lysate was centrifuged to remove cell debris and loaded into a column containing 1 ml of Ni^2+^ charged Chelating Sepharose™ (GE Healthcare). After extensive washing using the lysis buffer, supplemented with 20 mM imidazole, the protein was eluted from column by linear gradient of 80 to 300 mM imidazole. The protein was further purified by Superdex 200™ 10/300 GL (GE Healthcare), equilibrated with 30 mM Tris pH 8. 0, 150 mM NaCl. The His tagged-Hp0310 was eluted as a single peak, roughly corresponding to a tetramer and migrated as a single 37.5 kDa species on SDS-PAGE (theoretical mass: 33.7 Kda plus the 2.8 Kda His-tag).

To check the quality of the purified recombinant protein DLS and CD were performed (Figure 4).

### Crystallization and structure determination

The purified protein was concentrated to 20 mg/ml and used for crystallization trials, partially automated using an Oryx 8 crystallization robot (Douglas Instruments). The best crystals were obtained at 20°C by vapor diffusion technique using a 18 mg/ml protein stock solution and, as precipitant, a solution containing 0.2 M Ammonium Sulfate, 0.1 M Tri Sodium citrate pH 5.6, 15% (w/v) PEG 4000 (PEG II crystal screening solution no. 56 from Molecular Dimension, UK). Crystals could be processed as orthorhombic, space group C222_1_, with **a** = 154.56 Å, **b** = 154.73.44 Å, **c** = 158.68 Å. Since **a** and **b** cell parameters are very similar, data were also processed in a tetragonal crystal system, point groups P4 or P422, but in both cases the merging R factor was significantly higher (around 0.30), and the orthorhombic system was selected. The identity between two cell parameters can also induce merohedral twinning in the crystal, according to the law (*k,h,-l*). In the crystal used for structure determination and refinement the twinning fraction was estimated to be 0.19. One tetramer is present in the asymmetric unit, with VM = 4.13 Å^3^/Da corresponding to a solvent content of 70%. The high solvent content justifies the maximum resolution of 2.57 Å. The data set used in the final refinement was measured at the beamline BM30 of European Synchrotron Radiation Facility, Grenoble, France. It was indexed and integrated with Mosflm software [19] and merged and scaled with Scala [20], contained in the CCP4 crystallographic package [21]. The structure was solved by molecular replacement using Phaser software [22] starting from a tetrameric model built by the SWISS-PDB server using as a template the crystal structure of PuuE allantoinase (PDB ID code 3CL6 [11]). Refinement was carried on using the simulated annealing procedure contained in CNS [23] in the first stages of refinement and Refmac [24] and Phenix [25] in the subsequent steps. Several steps of manual rebuilding, performed with Coot graphic software [26], were necessary in order to reach the final structure, which significantly diverges from the starting model. Solvent molecules were added with the automated procedure of Phenix. Since the first cycles of refinement a maximum of about 14σ was clearly visible in the electron density map. Given the presence of two histidines and one aspartate residues coordinating it, it was interpreted as a Zn ion.

The final model contains 9429 protein atoms, four Zn atoms and 860 water molecules. The crystallographic R factor is 0.2056 (R_free_ 0.2350). Statistics on data collection and refinement are reported in Table 1. Geometrical parameters of the model, checked with Procheck [27], are as expected or better for this resolution. The coordinates and structure factors have been deposited at the Protein Data Bank with PDB ID code 3QBU. Buried surface calculations were performed using the Areaimol program [21].

**Table 1:** Data collection and refinement statistics.

Data collection[Table-fn nt102] *	
Space group	C222_1_
Cell dimensions	
***a***, ***b***, ***c*** (Å)	154.56, 154.73, 158.68
Resolution (Å)	69.54-2.57 (2.71-2.57)
*R*_sym_ or *R*_merge_	0.125 (0.505)
<*I*/σ(*I*)>	6.4 (2.0)
Completeness (%)	98.1 (97.6)
Redundancy	3.8 (3.7)

Refinement	
No. reflections	59318
*R*_work_/*R*_free_	0.2056/0.2350
No. atoms	
Protein	9429
Ion ligand	4
Water	860
R.m.s. deviations	
Bond lengths (Å)	0.007
Bond angles (°)	1.05
Ramachandran plot (%)	
favored	92.6
outliers	1.5
Overall G factor	0.1

A wavelength of 0.9765 Å was used. Rotations of 1° were performed.

*1 crystal was used to collect all diffraction data.

*Highest-resolution shell is shown in parentheses.

### Bioinformatics

Search and comparison of genetic clusters containing HP0310 and homologous gene were conducted using the Microbesonline web server (http://microbesonline.org). Sequence alignments were visualized with Espript [28] and carried out with Clustalw [29], with manual adjastements based on structure comparisons. Phylogenetic analysis was performed using the neighbor-joining method [30] implemented in Clustalw, and the resulting unrooted tree was visualized as radial layout with FigTree (http://tree.bio.ed.ac.uk). The absence of a signal peptide in the sequence of HP0310 and its close homologs was predicted using the SignalP server (http://www.cbs.dtu.dk/services/SignalP).

### Activity tests

Deacetylase activity of HP0310 in solution was assayed in three ways: for substrates without a free amino group before the reaction, a fluorogenic test was used; for all the others, except allantoin, by determining the amount of acetate liberated from the substrate during the reaction using an acetic acid kit (R-biopharm); allantoinase activity was determined by circular dichroism. All experiments were performed using the enzyme as purified from *E. coli* and after incubation of the enzyme with EDTA for 1 hour at room temperature to extract the ion bound and subsequent addition of different cofactors, as described below.

### Flurogenic Test

Enzymatic activity with N-acetyl glucosamine (GlcNAc), chitotriose GlcNAc3 and N-acetyl dipeptide (Ac-D-Ala-D-Ala-OH, Ac-D-Ala-D-Ala-OCH_3_, Ac-D-Ala-L-Ala-OH, Ac-D-Ala-D-Ala-OCH_3_) was performed according to [6], with slight modification. In brief, a reaction mixture consisting of 1 µM peptidoclycan deacetylase, 5 µM cofactor (ZnCl_2_, MgCl_2_, CoCl_2_, MnCl_2_ or NiSO_4_), 50 mM Bis·Tris (pH 7.5), and 2 mM substrate (Sigma) in a total volume of 1 ml, was incubated for 90 min at 37°C. 2 microliters of 0.4 M borate buffer (pH 9.0) was then added and free amines labeled with 3 µl of 2 mg/ml fluorescamine in DMSO for 15 min at room temperature. The labeling reaction was terminated by the addition of 3 µl of DMF/H2O (1∶1). Fluorescence was quantified by using an Ascent Fluorescence Reader (Thermo Electron Corporation, Finland), with excitation and emission wavelengths of 355 and 455 nm, respectively. The production of free amine was quantified with a glucosamine standard. All measurements were performed in triplicate.

### Acetate test

The reaction mixture (0.5 ml) contained 50 mM HEPES buffer (pH 7.0), 5 mM N-acetyl putrescine, 10 µM ZnCl_2_ and different amounts of the enzyme. The mixture was incubated at 37°C for 4 h and then boiled for 3 min to arrest the reaction. The release was determined with the acetic acid kit. The final calculations were performed by using the formula provided in the kit.

### Allontoinase activity

Enzymatic activity with Allantoin was performed according to [11]. Circular dichroism measurements were carried out at 25°C in a 10-mm path length cuvette with a Jasco J715 spectropolarimeter. HP0310 activity was determined using (R,S)-allantoin (0.5 mM) as substrates in the presence of HP0310 (0.70 µM) in 1 ml of PBS, pH 7.5. CD spectra were recorded in the 200–350 nm range for 10 min, one spectrum every min. The last spectrum was collected after 20 min.

## Supporting Information

Figure S1 Comparison of the HpPgdA genomic context in *Helicobacter pylori* and other bacteria. The analysis has been performed with the Microbesonline server (http://microbesonline.org) using HP0310 (**A**) or HP0312 (**B**) as anchor genes for the comparison. Homologous genes occurring at the same locus in different genomes are colored according to their homology group as indicated in the legend.
(PDF)

Figure S2 Analytical gel filtration of HpPgdA. Protein was purified by Superdex 200™ 10/300 GL (GE Healthcare), equilibrated with 30 mM Tris pH 8.0, 150 mM NaCl. His tagged-HP0310 eluted as a single peak, roughly corresponding to a tetramer: molecular mass estimated from analytical gel filtration is 144.54 kDa, in good agreement with the calculated one for the tetramer, 149.5 kDa.
(PDF)

## References

[1] Boneca IG, Dussurget O, Cabanes D, Nahori MA, Sousa S (2007). A critical role for peptidoglycan N-deacetylation in listeria evasion from the host innate immune system.. Proc Natl Acad Sci U S A.

[2] Strober W, Murray PJ, Kitani A, Watanabe T (2006). Signalling pathways and molecular interactions of NOD1 and NOD2.. Nat Rev Immunol.

[3] Meylan E, Tschopp J, Karin M (2006). Intracellular pattern recognition receptors in the host response.. Nature.

[4] Wang G, Maier SE, Lo LF, Maier G, Dosi S (2010). Peptidoglycan deacetylation in *Helicobacter pylori* contributes to bacterial survival by mitigating host immune responses.. Infect Immun.

[5] Wang G, Olczak A, Forsberg LS, Maier RJ (2009). Oxidative stress-induced peptidoglycan deacetylase in *Helicobacter pylori*.. J Biol Chem.

[6] Blair DE, Schuttelkopf AW, MacRae JI, van Aalten DM (2005). Structure and metal-dependent mechanism of peptidoglycan deacetylase, a streptococcal virulence factor.. Proc Natl Acad Sci U S A.

[7] Franco AT, Friedman DB, Nagy TA, Romero-Gallo J, Krishna U (2009). Delineation of a carcinogenic *Helicobacter pylori* proteome.. Mol Cell Proteomics.

[8] Banner DW, Bloomer A, Petsko GA, Phillips DC, Wilson IA (1976). Atomic coordinates for triose phosphate isomerase from chicken muscle.. Biochem Biophys Res Commun.

[9] Blair DE, van Aalten DM (2004). Structures of bacillus subtilis PdaA, a family 4 carbohydrate esterase, and a complex with N-acetyl-glucosamine.. FEBS Lett.

[10] Holm L, Sander C (1993). Protein-structure comparison by alignment of distance matrices.. J Mol Biol.

[11] Ramazzina I, Cendron L, Folli C, Berni R, Monteverdi D (2008). Logical identification of an allantoinase analog (PuuE) recruited from polysaccharide deacetylases.. J Biol Chem.

[12] Luthy L, Grutter MG, Mittl PR (2002). The crystal structure of *Helicobacter pylori* cysteine-rich protein B reveals a novel fold for a penicillin-binding protein.. J Biol Chem.

[13] Dickmanns A, Ballschmiter M, Liebl W, Ficner R (2006). Structure of the novel alpha-amylase AmyC from *Thermotoga maritima*.. Acta Crystallogr D Biol Crystallogr.

[14] Kamio Y, Nakamura K (1987). Putrescine and cadaverine are constituents of peptidoglycan in *Veillonella alcalescens* and *Veillonella parvula*.. J Bacteriol.

[15] Tokuyasu K, Mitsutomi M, Yamaguchi I, Hayashi K, Mori Y (2000). Recognition of Chitooligosaccharides and Their *N*-Acetyl Groups by Putative Subsites of Chitin Deacetylase from a Deuteromycete, *Colletotrichum lindemuthianum*.. Biochemistry.

[16] Hekmat O, Tokuyasu K, Withers SG (2003). Subsite structure of the endo-type chitin deacetylase from a Deuteromycete, *Colletotrichum lindemuthianum*: an investigation using steady-state kinetic analysis and MS.. Biochem J.

[17] Smith TG, Lim JM, Weinberg MV, Wells L, Hoover TR (2007). Direct analysis of the extracellular proteome from two strains of *Helicobacter pylori*.. Proteomics.

[18] Patti GJ, Chen J, Schaefer J, Gross ML (2008). Characterization of structural variations in the peptidoglycan of vancomycin-susceptible *Enterococcus faecium*: Understanding glycopeptide-antibiotic binding sites using mass spectrometry.. J Am Soc Mass Spectrom.

[19] Leslie AGW (2006). The integration of macromolecular diffraction data.. Acta Crystallogr D Biol Crystallogr.

[20] Evans P (2006). Scaling and assessment of data quality.. Acta Crystallogr D Biol Crystallogr.

[21] Collaborative Computational Project, Number 4. (1994). The CCP4 suite: Programs for protein crystallography.. Acta Crystallogr D Biol Crystallogr.

[22] McCoy AJ, Grosse-Kunstleve RW, Adams PD, Winn MD, Storoni LC (2007). Phaser crystallographic software.. J Appl Crystallogr.

[23] Brunger AT, Adams PD, Clore GM, DeLano WL, Gros P (1998). Crystallography & NMR system: A new software suite for macromolecular structure determination.. Acta Crystallogr Sect D-Biol Crystallogr.

[24] Murshudov GN, Vagin AA, Dodson EJ (1997). Refinement of macromolecular structures by the maximum-likelihood method.. Acta Crystallogr Sect D-Biol Crystallogr.

[25] Adams PD, Afonine PV, Bunkoczi G, Chen VB, Davis IW (2010). PHENIX: A comprehensive python-based system for macromolecular structure solution.. Acta Crystallogr D Biol Crystallogr.

[26] Emsley P, Cowtan K (2004). Coot: Model-building tools for molecular graphics.. Acta Crystallogr Sect D-Biol Crystallogr.

[27] Laskowski RA, Macarthur MW, Moss DS, Thornton JM (1993). Procheck - a program to check the stereochemical quality of protein structures.. J Appl Crystallogr.

[28] Gouet P, Courcelle E, Stuart DI, Metoz F (1999). ESPript: Analysis of multiple sequence alignments in PostScript.. Bioinformatics.

[29] Thompson JD, Higgins DG, Gibson TJ (1994). CLUSTAL W: Improving the sensitivity of progressive multiple sequence alignment through sequence weighting, position-specific gap penalties and weight matrix choice.. Nucleic Acids Res.

[30] Saitou N, Nei M (1987). The neighbor-joining method: A new method for reconstructing phylogenetic trees.. Mol Biol Evol.

[31] Delano WL (2008). The PyMOL Molecular Graphics System.

