# Supplementary material for: The Structure of Helicobacter pylori HP0310 Reveals an Atypical Peptidoglycan Deacetylase
Source: PLoS One. 2011 Apr 29;6(4):e19207. doi: 10.1371/journal.pone.0019207 (PMC3084791; doi:10.1371/journal.pone.0019207)
Supplement: Figure S1 — Comparison of the HpPgdA genomic context in Helicobacter pylori and other bacteria. The analysis has been performed with the Microbesonline server (http://microbesonline.org) using HP0310 (A) or HP0312 (B) as anchor genes for the comparison. Homologous genes occurring at the same locus in different genomes are colored according to their homology group as indicated in the legend. (PDF) [file pone.0019207.s001.pdf]

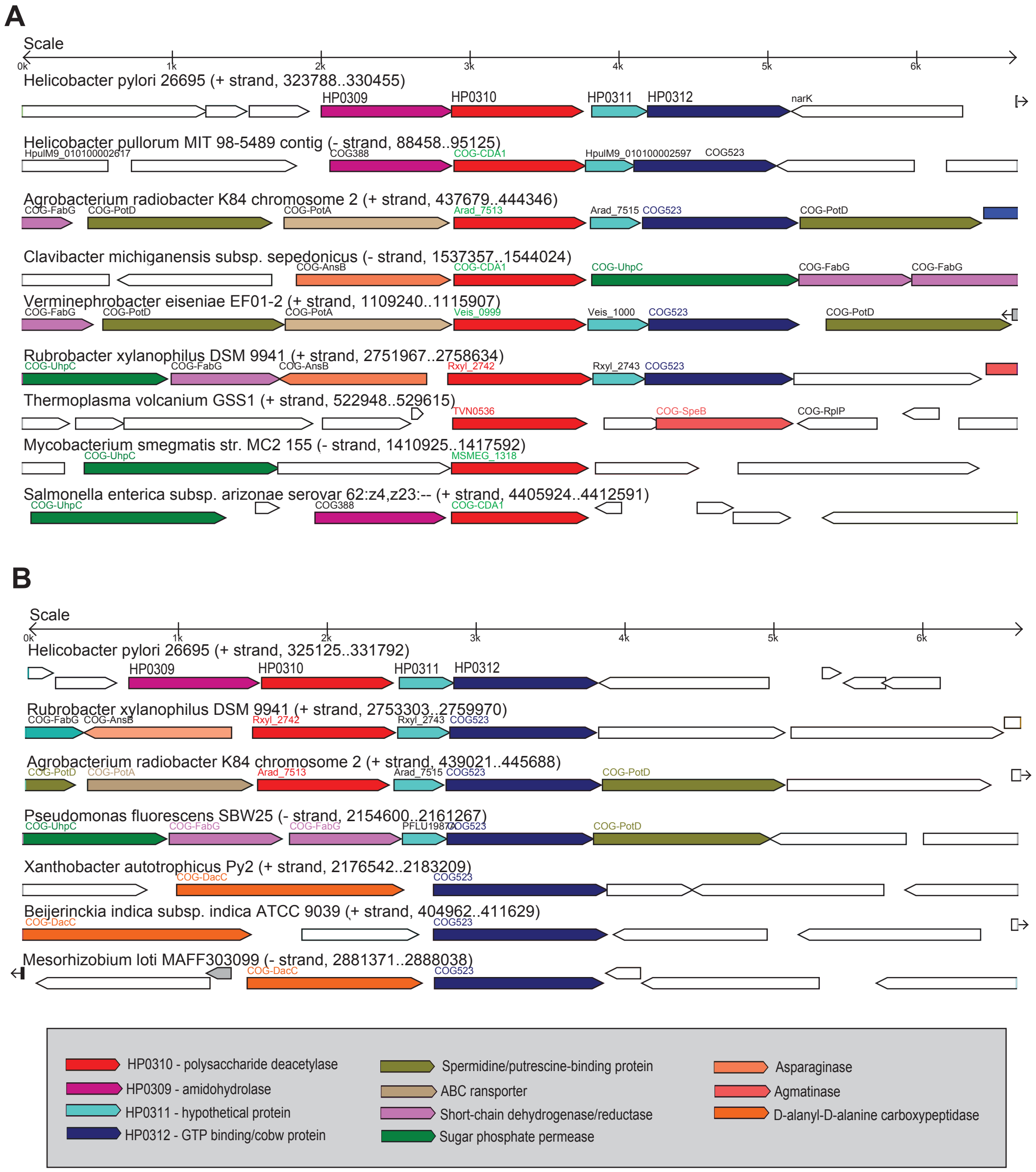

Supplementary Figure S1. Comparison of the HP0310 genomic context in *Helicobacter pylori* and other bacteria. The analysis has been performed with the Microbesonline server (http://microbesonline.org) using HP0310 (**A**) or HP0312 (**B**) as anchor genes for the comparison. Homologous genes occurring at the same locus in different genomes are colored according to their homology group as indicated in the legend.
