# Supplementary material for: The Structure of Helicobacter pylori HP0310 Reveals an Atypical Peptidoglycan Deacetylase
Source: PLoS One. 2011 Apr 29;6(4):e19207. doi: 10.1371/journal.pone.0019207 (PMC3084791; doi:10.1371/journal.pone.0019207)
Supplement: Figure S2 — Analytical gel filtration of HpPgdA. Protein was purified by Superdex 200™ 10/300 GL (GE Healthcare), equilibrated with 30 mM Tris pH 8.0, 150 mM NaCl. His tagged-HP0310 eluted as a single peak, roughly corresponding to a tetramer: molecular mass estimated from analytical gel filtration is 144.54 kDa, in good agreement with the calculated one for the tetramer, 149.5 kDa. (PDF) [file pone.0019207.s002.pdf]

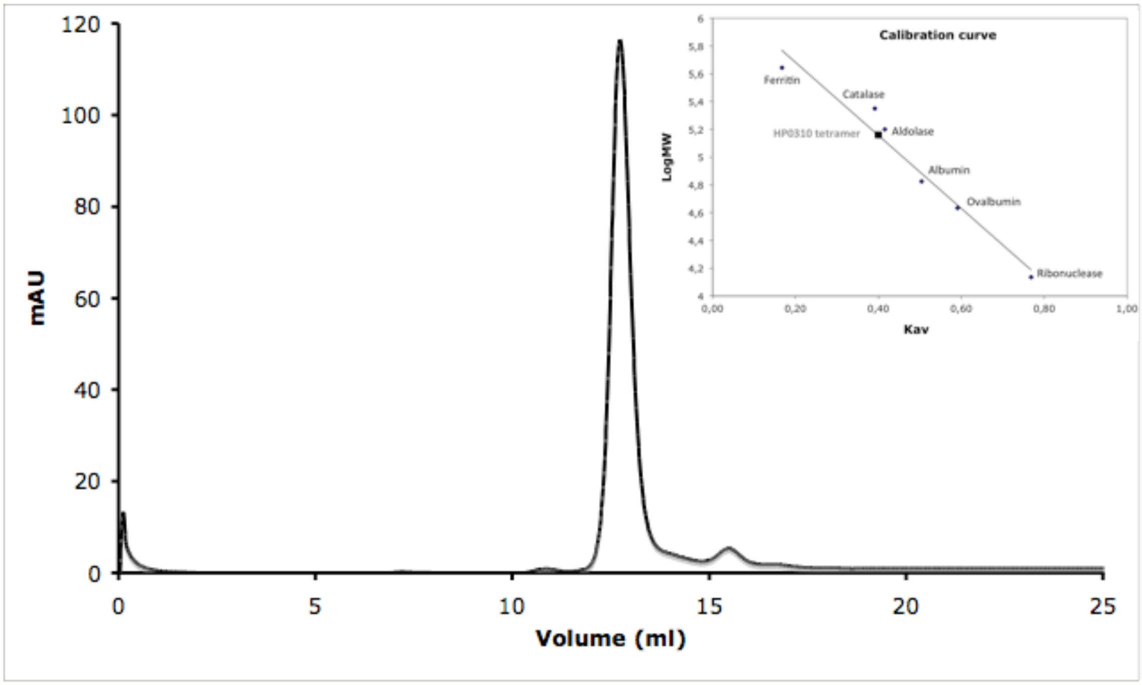

Supplementary Figure S2. Analytical gel filtration of HP0310. Protein was purified by Superdex 200™ 10/300 GL (GE Healthcare), equilibrated with 30 mM Tris pH 8.0, 150mM NaCl. His tagged-HP0310 eluted as a single peak, roughly corresponding to a tetramer: molecular mass estimated from analytical gel filtration is 144.54 kDa, in good agreement with the calculated one for the tetramer, 149.5 kDa.
